# Cognitive Factors Influencing Utterance Fluency in L2 Dialogues: Monadic and Non-monadic Perspectives

**DOI:** 10.3389/fpsyg.2022.926367

**Published:** 2022-06-30

**Authors:** Ruiling Feng

**Affiliations:** ^1^Foreign Language College, Tianjin Normal University, Tianjin, China; ^2^Department of Foreign Languages and Literatures, Tsinghua University, Beijing, China

**Keywords:** utterance fluency in L2 dialogues, interlocutor, monadic perspective, non-monadic perspective, cognitive factors

## Introduction

Studies of second language (L2) speech fluency have largely focused on monologs, while dialogues are rarely studied (McCarthy, 2010; Tavakoli, 2016; Foster, 2020). In dialogues, two or more interlocutors take turns contributing to the flow of interaction. Therefore, utterance fluency (overt fluency performance) in L2 dialogues, including individual/within-turn fluency and interactional/between-turn fluency (Peltonen, 2017a, 2020), requires not only speaker-internal cognitive processing but also between-speaker cognitive cooperation based on shared understanding (Roever and Kasper, 2018; Pickering and Garrod, 2021). Speaker-internal cognitive factors are activated by social interaction (Tavakoli and Wright, 2020). Consequently, examination of cognitive factors influencing utterance fluency in L2 dialogues should include both a monadic perspective that hinges on each individual's private cognitive processing and a non-monadic perspective that analyzes the dialogue as a whole system by considering the relationship between each individual's utterances (Tavakoli and Wright, 2020; Pickering and Garrod, 2021). Incorporating both perspectives could contribute to the ongoing discussion about factors influencing L2 speech production. Especially, the non-monadic view can help reconcile the overreliance on individual fluency performance. Therefore, this paper aims to examine a tentative list of cognitive factors affecting utterance fluency in L2 dialogues from the two perspectives.

## Monadic Perspective

The monadic perspective focuses on factors affecting speaker-internal mental activities. From this perspective, cognitive factors influencing fluency in L2 dialogues mainly include L2-specific cognitive fluency (access to L2 knowledge), general cognitive fluency (reflected in personal speaking style), and overall L2 proficiency (linguistic repertoire) (e.g., Segalowitz, 2010, 2016; Kahng, 2014, 2020; Pérez Castillejo, 2018).

### L2-Specific Cognitive Fluency

Studies relating L2-specific cognitive fluency to L2 utterance fluency are reviewed in this section. Segalowitz (2010) argued that L2 fluency performance is influenced by both L2-specific cognitive fluency and language-independent personal speaking style. His argument has been corroborated by later studies (e.g., Kahng, 2014, 2020; Segalowitz, 2016; Suzuki and Kormos, 2022). L2-specific cognitive fluency is gained by partialling out first language (L1) data from equivalent L2 data (Segalowitz, 2010, 2016; Bradlow et al., 2017). Cognitive processes could consist of the four cognitive modules in Levelt's (1989; 1999) speech production model, including conceptualization (preverbal message generation), formulation (grammatical and morpho-phonological encoding), articulation, and monitoring (self-perception) (Tavakoli et al., 2020).

Regarding L2-specific cognitive fluency, the four cognitive modules have received uneven scholarly attention. Conceptualization is regarded as language-independent (e.g., Levelt, 1989, 1999; De Bot, 1992; Segalowitz, 2010); therefore, it is generally excluded from this research strand. Among the other three modules, formulation and articulation are the main foci, formulation in particular. Segalowitz and Freed (2004) measured the L2-specific speed of lexical access and attention control by the reaction time in a semantic classification task and the efficiency of the two measures by the coefficient variation (standard deviation divided by the mean) of the reaction time. Segalowitz (2016) also adopted reaction time and coefficient variation of it, and linguistic attention flexibility. Different from Segalowitz's tests, Kahng (2020) measured lexical retrieval by a picture-naming task and syntactic encoding by a sentence completion task. Besides the aforementioned quantitative measurement, qualitative measurement of formulation fluency is also used. For example, Kahng (2014) adopted stimulated recall to tap thoughts during filled and silent pauses. As for the measurement of articulation, delayed picture naming tasks (e.g., de Jong and Mora, 2017; Kahng, 2020) and controlled speech tasks (Suzuki and Kormos, 2022) have been employed. Monitoring is rarely studied as a dimension of L2-specific cognitive fluency or in terms of its relationship to utterance fluency. However, some monitoring-related features (e.g., repetitions and self-corrections) have been found language-independent rather than L2-specific (Peltonen and Lintunen, 2016; Georgiadou and Roehr-Brackin, 2017; Olkkonen, 2017).

### General Cognitive Fluency

Recent studies have found that equivalent L1 fluency performance measures, accounting for general cognitive fluency or stable personal speaking style, can help explain L2 utterance fluency (e.g., Segalowitz, 2010, 2016; Bradlow et al., 2017; Kahng, 2020). For example, mean silent pause duration and filled pause frequency are mainly related to general instead of L2-specific cognitive fluency (de Jong et al., 2013; Kahng, 2020). General cognitive fluency is especially associated with conceptualization as encyclopedia knowledge rather than linguistic knowledge is used in this stage (Segalowitz, 2010; Kahng, 2014, 2020). However, not all L2 fluency performance measures demonstrate a correlation with general cognitive fluency. For instance, L2 speech rate change cannot be predicted by the equivalent L1 measure, and therefore might be an L2-specific feature (Baese-Berk and Morrill, 2015; Baese-Berk and Bradlow, 2021). Besides, overall L2 proficiency could moderate the relationship between general cognitive fluency and L2 utterance fluency, as speakers of higher proficiency demonstrate a stronger correlation between L1 and L2 utterance fluency (Huensch and Tracy–Ventura, 2017; Peltonen, 2018). However, overall L2 proficiency does not mediate the relationship (Duran-Karaoz and Tavakoli, 2020).

### Overall L2 Proficiency

Overall L2 proficiency represents L2 linguistic repertoire and influences fluency performance in L2 dialogues (Kahng, 2020; Tavakoli and Wright, 2020). L2 and L1 speakers demonstrate different (dis)fluency patterns. For example, L2 speakers pause markedly more and longer within clauses than L1 speakers do (Kahng, 2014; de Jong, 2016). It could be that L2 speakers are under higher processing time pressure (Baddeley, 2003), due to smaller processing units and lower automaticity (Kroll and de Groot, 1997; Jiang, 2000; Bundgaard-Nielsen et al., 2011; Wang, 2014; Tavakoli and Wright, 2020).

Some fluency-related studies have examined L2 speakers of different proficiency levels. With higher proficiency, reliance on L1 mediation decreases in L2 lexical retrieval (Jiang, 2000), leading to greater processing automaticity with lower switching costs (Costa and Santesteban, 2004; DeKeyser, 2005; Segalowitz, 2010). Williams and Korko (2019) found advanced speakers showed fewer corrections, silent pauses, and filled pauses than lower intermediate speakers in L2 monologs. They attributed the differences to automaticity and the use of formulaic structures. Besides, proficiency affects the length and frequency of turn pauses in dialogues (Peltonen, 2017b; van Os et al., 2020). Lower-proficiency speakers are more hesitant to start turns, resulting in longer and more turn pauses (Peltonen, 2017b), while higher-proficiency speakers could be better ready to take turns with higher automaticity.

## Non-monadic Perspective

The non-monadic perspective analyzes dialogue as a whole system with interaction and interdependence between interlocutors (McCarthy, 2010; Segalowitz, 2016; Tavakoli, 2016; Peltonen, 2017b; Tavakoli and Wright, 2020). As such, speaker stance, interactional competence, and interlocutors' cognitive factors are viewed as potentially contributing to fluency in L2 dialogues.

### Speaker Stance

Speaker stance represents an attitude, willingness, or orientation instead of ability and can affect how individuals engage in dialogues. If speakers regard dialogue as a self-performing activity and take a safer speaker stance, they would pay substantial attention to their own production but little to interlocutors' utterances (He and Dai, 2006; Tavakoli and Wright, 2020; Pickering and Garrod, 2021). A safer speaker stance might help achieve higher within-turn fluency, however, at the sacrifice of interactive listening and contingent responses. In contrast, a more other-oriented speaker might consider dialogue as a joint activity and keep both speaker and listener roles active concurrently (Pickering and Garrod, 2021). Therefore, other-oriented speakers are more inclined to incorporate interactive listening, between-turn responsiveness, and between-speaker alignment and synchrony. Compared to a safer speaker stance, a more other-orientated stance may slow down one's speech production due to more time for comprehension and hence less time for production (Tavakoli and Wright, 2020). The relationship between speaker stance and fluency in L2 dialogues might be moderated by overall L2 proficiency, which affects attentional resources allocated to individual speech and interactional aspects of dialogues (Levelt, 1989; Kormos, 2006).

Introspective and retrospective self-assessment can detect speaker stance, exploring speakers' perceptions of and attitudes toward speech tasks (Alderson, 1985). For example, He and Dai (2006) designed a questionnaire to tap how students viewed and dealt with the group discussion in a high-stakes test. Results showed that most students took a safer speaker stance, largely attending to processing individual turns rather than listening and responding to interlocutors. As such, they could display their most fluent English with long turns but low responsiveness to the just-uttered turn from interlocutors. The safer speaker stance could be associated with factors such as culture and specific task context (e.g., high-stakes tests vs. free discussions).

### Interactional Competence

Interactional competence refers to one's ability to adopt different communication strategies, and actively listen and respond to previous speakers' contributions based on proper comprehension (Galaczi, 2014; May et al., 2019). It is important for the co-construction of dialogues (Roever and Kasper, 2018) and affects both individual/within-turn and interactional/between-turn fluency (May et al., 2019; Tavakoli and Wright, 2020). Speakers of higher interactional competence are more likely to respond to and synchronize with interlocutors, while those of lower competence might experience difficulties engaging in dialogues as they cannot guarantee appropriate responsiveness and synchrony (Galaczi, 2008). Synchronization could help keep interlocutors on the same wavelength (Ward and Tsukahara, 2003), and increase fluency in a dialogue as a whole system instead of an individual performance (Pickering and Garrod, 2021). Note that interactional competence might overlap with fluency in dialogues in features like turn pause and breakdown repair (Galaczi and Taylor, 2018; Zhang and Jin, 2021).

Interactional competence is difficult to operationalize due to its multicomponential nature (Galaczi, 2014). Here I propose two dimensions for the measurement of interactional competence, interactive listening and between-turn responsiveness (e.g., May, 2009, 2011; Lam, 2018; Ross, 2018). Interactive listening represents attention to interlocutors' utterances. It aims to show support and comprehension. Responsiveness between adjacent turns could promote predictability of the dialogic flow, and thus fluency (Smith and McMurray, 2018; Pickering and Garrod, 2021). These two dimensions are inevitably related in dialogues, as producing a turn contingent on the just-uttered turn depends on comprehension as a result of interactive listening, though interactive listening cannot guarantee comprehension or responsiveness (Galaczi, 2014).

Interactive listening can be measured by verbal and non-verbal features. Verbal features include listener support moves such as backchannelling and confirmation of comprehension (Galaczi, 2014; Lam, 2018). Non-verbal features refer to paralinguistic features like eye contact and gesticulation (Jenkins and Parra, 2003; Ross, 2018). These features signal listener attentiveness but not necessarily comprehension (Ross, 2018). Sometimes, they are even used to mask insufficient comprehension (Galaczi, 2014; Lam, 2018).

Adequate between-turn responsiveness can demonstrate a link to and extension of the previous speaker's contribution (Galaczi, 2014). A responsive turn (contingent response in Lam, 2018) may include three conversational actions, namely formulation of a just-uttered turn, explaining (dis)agreement with the previous turn, and expanding the topic (Lam, 2018). Based on these actions, Lam proposed three proficiency levels of producing responsive turns (lower, mid, and higher levels).

### Interlocutors' Cognitive Factors

In dialogues, interlocutors tend to align and synchronize with each other (Pickering and Garrod, 2021); therefore, the aforementioned cognitive factors of each interlocutor may impact, indirectly *via* their utterances, other interlocutors' fluency performance (Tavakoli and Wright, 2020; Pickering and Garrod, 2021). Speakers' competence and performance in a dialogue can decide, to a large extent, how and what their interlocutors try to comprehend, respond to, and align and synchronize with (Benuš, 2021; Pickering and Garrod, 2021). Previous studies have found dialogue partners converge in some fluency features, for example, inter-word intervals (Himberg et al., 2015) and speech rate (Cohen Priva et al., 2017). The synchronization could facilitate more seamless turn switching (Pickering and Garrod, 2021). Even highly self-oriented speakers have to synchronize somehow with and therefore be influenced by their interlocutors.

## Conclusions

Analysis of cognitive factors affecting utterance fluency in L2 dialogues should incorporate both monadic and non-monadic perspectives. Monadically, L2-specific cognitive fluency, general cognitive fluency, and overall L2 proficiency could affect speakers' fluency performance *via* private cognitive processing. Non-monadically, speaker stance, interactional competence, and interlocutors' cognitive factors influence how speakers listen to, comprehend, and accommodate interlocutors' utterances, and therefore their fluency performance in L2 dialogues. Cautions should be made when predicting utterance fluency in L2 dialogues with a myriad of cognitive factors. For example, some factors might be correlated, which leads to multicollinearity. Also, a linear relationship might not exist between fluency and some factors such as proficiency. This paper focuses on cognitive factors, while affective and sociocultural factors could also affect fluency (Sun and Zhang, 2020; Sun, 2022). These factors warrant future research from monadic and non-monadic perspectives regarding utterance fluency in L2 dialogues.

## Author Contributions

The author confirms being the sole contributor of this work and has approved it for publication.

## Funding

This work was supported by the Project of Discipline Innovation and Advancement (PODIA)-Foreign Language Education Studies at Beijing Foreign Studies University (Grant Number: 2020SYLZDXM011), Beijing.

## Conflict of Interest

The author declares that the research was conducted in the absence of any commercial or financial relationships that could be construed as a potential conflict of interest.

## Publisher's Note

All claims expressed in this article are solely those of the authors and do not necessarily represent those of their affiliated organizations, or those of the publisher, the editors and the reviewers. Any product that may be evaluated in this article, or claim that may be made by its manufacturer, is not guaranteed or endorsed by the publisher.
